# Exploring the use and challenges of implementing virtual visits during COVID-19 in primary care and lessons for sustained use

**DOI:** 10.1371/journal.pone.0253665

**Published:** 2021-06-24

**Authors:** Heba Tallah Mohammed, Lirije Hyseni, Victoria Bui, Beth Gerritsen, Katherine Fuller, Jihyun Sung, Mohamed Alarakhia

**Affiliations:** 1 eHealth Centre of Excellence, Kitchener, Ontario, Canada; 2 Hamilton Family Health Team, Hamilton, Ontario, Canada; 3 Department of Medicine, McMaster University, Hamilton, Ontario, Canada; 4 Department of Family Medicine, McMaster University, Hamilton, Ontario, Canada; Faculty of Health Sciences - Universidade da Beira Interior, PORTUGAL

## Abstract

**Background:**

The COVID-19 pandemic has rapidly transformed how healthcare is delivered to limit the transmission of the virus. This descriptive cross-sectional study explored the current use of virtual visits in providing care among primary care providers in southwestern Ontario during the first wave of the COVID-19 pandemic and the anticipated level of utilization post-pandemic. It also explored clinicians’ perceptions of the available support tools and resources and challenges to incorporating virtual visits within primary care practices.

**Methods:**

Primary care physicians and nurse practitioners currently practicing in the southwestern part of Ontario were invited to participate in an online survey. The survey invite was distributed via email, different social media platforms, and newsletters. The survey questions gathered clinicians’ demographic information and assessed their experience with virtual visits, including the proportion of visits conducted virtually (before, during the pandemic, and expected volume post-pandemic), overall satisfaction and comfort level with offering virtual visits using modalities, challenges experienced, as well as useful resources and tools to support them in using virtual visits in their practice.

**Results:**

We received 207 responses, with 96.6% of respondents offering virtual visits in their practice. Participants used different modalities to conduct virtual visits, with the vast majority offering visits via phone calls (99.5%). Since the COVID-19 pandemic, clinicians who offered virtual visits have conducted an average of 66.4% of their visits virtually, compared to an average of 6.5% pre-pandemic. Participants anticipated continuing use of virtual visits with an average of 43.9% post-pandemic. Overall, 74.5% of participants were satisfied with their experience using virtual visits, and 88% believed they could incorporate virtual visits well within the usual workflow. Participants highlighted some challenges in offering virtual care. For example, 58% were concerned about patients’ limited access to technology, 55% about patients’ knowledge of technology, and 41% about the lack of integration with their current EMR, the increase in demand over time, and the connectivity issues such as inconsistent Wi-Fi/Internet connection. There were significant differences in perception of some challenges between clinicians in urban vs, rural areas. Clinicians in rural areas were more likely to consider the inconsistent Wi-Fi and limited connectivity as barriers to incorporating virtual visits within the practice setting (58.8% vs. 40.2%, P = 0.030). In comparison, clinicians in urban areas were significantly more concerned about patients overusing virtual care services (39.4% vs. 21.6%, P = 0.024). As for support tools, 47% of clinicians advocated for virtual care standards outlined by their profession’s college. About 32% identified change management support and technical training as supportive tools. Moreover, 39% and 28% thought local colleagues and in-house organizational support are helpful resources, respectively.

**Conclusion:**

Our study shows that the adoption of virtual visits has exponentially increased during the pandemic, with a significant interest in continuing to use virtual care options in the delivery of primary care post-pandemic. The study sheds light on tools and resources that could enhance operational efficiencies in adopting virtual visits in primary care settings and highlights challenges that, when addressed, can expand the health system capacity and sustained use of virtual care.

## Introduction

The advancement of information technology has allowed for incorporating digital infrastructure within the healthcare system [1]. Digital tools can improve access to healthcare services and enhance care delivery [2]. One of the leading opportunities is virtual care [1, 3]. Using various communication tools or information technologies, clinicians can interact with their patients within their circle of care remotely, enabling a virtual visit [4]. Different modalities are frequently used to conduct virtual visits, supporting both synchronous and asynchronous communication between healthcare providers and patients. Such modalities include telephone calls and video conferencing (synchronous), which require both the patient and the healthcare provider to be engaged at the same time, and digital messaging modalities such as secure messaging, e-mail, and text messaging, supporting asynchronous communications [1, 5]. For any venue, virtual care requests can be initiated by patients for any non-urgent service [6].

Out of the 85.5 million virtual contacts covered under the Kaiser Permanente System health plan in the US, 50% were done by phone, 40% through secure messaging and 0.2% video visits [4, 7]. However, a large study conducted in 2020 evaluating 14,317 virtual care visits in Canada reported that both patients and clinicians are more inclined to use asynchronous communication in the form of secure messaging for their virtual care visits [6]. To support the uptake of virtual care within the Canadian healthcare settings, governmental agencies such as Ontario Health (Ontario Telemedicine Network division) and private corporations such as Novari Health (Kingston, Ontario, Canada), Think Research (Toronto, Ontario, Canada), Google (Mountain View, California, United States), Apple (Cupertino, California, United States), Microsoft (Redmond, Washington, United States), Zoom (San Jose, California, United States), and others are providing various platforms. Virtual care platforms may be integrated within clinicians’ electronic medical records (EMR) or non-integrated [3, 6].

Evidence shows that virtual care opens new possibilities to overcome barriers for patients who have difficulty attending in-person care and ensures equity in accessing care [1, 3]. For patients with limited access to transportation, mobility impairments, or residents in a long-term care setting, this modality of care allows them to continue receiving medical guidance and support managing their conditions [3, 5]. Overall, the use of virtual visits has been associated with high patient satisfaction, better access to care, and overall cost savings [3]. Many studies have documented a satisfactory experience for patients and providers when using virtual care [3, 8, 9]. For example, an observational study conducted in British Columbia to assess patients’ opinion of virtual visits reported that about 93% of patients had a positive experience with virtual visits, and 91% described it as helpful to resolve their health issues [1]. Other studies have reported similar opinions on the reasonable care provided by virtual visits across different services such as orthopedic surgery, trauma management and minor injury treatment, where digital imaging tools can provide supplementary information [3, 9]. Besides the considerable interest in using virtual services among patients and clinicians [3, 4, 9], incorporating virtual care within clinical workflow has also shown some economic benefits to health care settings [9]. A study conducted in Australia reported a 32% return on investment to rural health care settings when applying a virtual health care model [9].

Integrated Care is defined as the organization of care around an individual patient where the care providers collaborate across sectors to achieve the best possible outcome for the patient [10]. In 2019, Ontario Health Teams (OHTs) were introduced in Ontario to organize and deliver as an integrated care network [2]. To achieve this vision, OHTs are expected to adopt digital health technologies to support patient-centred care [2]. Within the first year, OHTs are expected to expand virtual care offering to 2–5% of their Year 1 population [2]. However, clinicians’ experience with using digital tools was limited [3, 11]. A survey conducted in 2018 by Canada Health Infoway reported a gap between the number of virtual services provided by clinicians and patients’ demands for electronic access to services [4]. Fundamental barriers to adoption include ambiguity about remuneration, privacy and security, virtual visits’ efficacy compared to in-person care, technological literacy, availability of technical support, and hardware and administrative resources [3, 12].

The COVID-19 pandemic has challenged the healthcare system by rallying various sectors to provide more integrated care through technology. It has had a transformational impact on the way healthcare is being delivered [11]. The need for virtual visits and other digital health tools has dramatically increased in order to limit the transmission of the virus and relieve an overwhelmed system [11]. Front-line workers have been pressured to quickly adopt digital tools to safely and effectively provide patient care [11]. In Ontario, primary care providers have been advised to “implement a system for virtual and telephone consultations as a preferred option, when and where possible [13].” This has led to a rapid shift in virtual care adoption whenever feasible.

In 2018–2019, clinicians conducted about 8% of their clinical visits virtually in Canada [4]. In 2020, with the COVID-19 pandemic, this percentage increased about 7.5-fold as clinicians conducted approximately 60% of their consults with patients virtually [14]. Despite the shift in the adoption and use of digital tools to reduce the risk of virus transmission, there is limited data that explores the extent of the current practice of virtual care during the pandemic, the challenges, and what is needed to make virtual care sustainable.

Given the rising expectations to adopt virtual care, a thorough understanding of primary care clinicians’ perspectives and the various challenges they face to adopting virtual care is necessary, especially as virtual care looks to become a standard part of care after the COVID-19 pandemic. This will help inform the digital strategy to support integrated care as it matures. To our knowledge, no published studies have assessed primary care providers’ perspectives on useful tools and resources to support the adoption of virtual care in Canada.

This study aimed to explore the current use of virtual visits in the delivery of care among primary care providers in the southwestern part of Ontario (known as the Ontario Health West Region) during the first wave of the COVID-19 pandemic and the anticipated level of utilization post-pandemic. The study also sought to understand the primary care providers’ perceptions of the available support tools and resources. Further, it aimed to identify factors that influence the success and the challenges to adopting and incorporating virtual visits within their practice.

## Material and methods

### Study design and population

This is a descriptive cross-sectional survey of community primary care practitioners—primary care physicians and nurse practitioners—currently practicing in the southwestern part of Ontario. An estimated 3379 [15] primary care physicians [16] and approximately 560 nurse practitioners practice in primary care within the region.

An email invite that included an electronic link to the survey was shared by one of the co-authors (MA)—the study principal investigator—with the Ontario Health Team digital leads within southwestern Ontario. The region leads then sent email invites to approximately 679 of their registered lists of primary care providers on August 16, 2020. The invite was also distributed on August 31, 2020, through the eHealth Centre of Excellence (eCE) Newsletter with the 435 existing primary care provider subscribers. Advertisements for the study were also publicized on the eCE Twitter and LinkedIn accounts. The eCE Twitter and LinkedIn accounts have 936 and 535 followers, respectively. Through the various recruitment methods used (newsletter, Twitter, LinkedIn and email invites), primary care providers were encouraged to share the email invite or study advertisement that included the link with others in their networks that may have been eligible to participate in the study. All participants were directed to review a consent form that described the different aspects of the research study, including the study’s objectives, eligibility requirements, expectations when taking part in the survey, any foreseeable risks, potential benefits, and how participation is voluntary.

A reminder email was sent to the list of primary care providers described above two weeks after the initial invite. A final reminder email through the eCE Newsletter was sent to subscribers again on September 30, 2020. The survey link remained active for seven weeks (August 17, 2020- October 5–2021) and directed the participants to the Survey Monkey platform, an online survey development cloud-based software.

#### Selection criteria

In general, the study’s invite advised potential participants that only primary care physicians and nurse practitioners practicing within Ontario Health West Region were eligible. When potential participants clicked on the survey link, they were first screened through a filter question regarding their role in their practice settings. Only those who identified primary care as their practice setting within Ontario Health West Region were eligible to participate. If the participants were not practicing at a primary care setting within the region, they were directed to the end of the survey. Data were collected from August 17, 2020, to October 5, 2020.

Due to the nature of outreach to eligible participants (described above) and the overlapping of subscribers and followers to the eCE different accounts (Newsletter, Twitter and LinkedIn), it has been challenging to determine an accurate number of primary care providers who received the invite. Of the potential participants who were approached, a total of 207 primary care practitioners responded and completed the survey.

### Data collection tool

A literature search on virtual care and digital tools, providers’ satisfaction, challenges, and available support was conducted [1, 11, 17–21]. Relevant survey questions and information related to the study’s objectives were used to build the survey instrument. With permission, some questions were adopted and modified from an online survey distributed amongst primary care physicians, nurse practitioners, and physician assistants in Hamilton, Ontario, earlier in 2020. [22]. Survey face and content validity were determined through extensive multiple separate review sessions among the authors.

Furthermore, the authors sought feedback from eight primary care clinicians (six family physicians and two nurse practitioners) practising in the Ontario Health West region. The questionnaire was modified accordingly, then further pilot-tested for question-wording, question structure, physical layout, length of time for survey completion, instructions, readability and reliability by the eight primary care providers. Further modification and formatting were done based on the clinicians’ feedback.

The final version of the survey consisted of 36 questions, 19 of which focused on virtual visits and demographic information and an additional 17 questions assessed participants’ perspectives and experience with fax and other digital tools. As this article focuses on clinicians’ experience with virtual visits, only data directly related to virtual visits and electronic medical records (EMRs) is presented in this article. A copy of the questionnaire is included in the supporting documents.

The questions focused on virtual visits consisted of questions that explored clinicians’ experience with virtual visits and their tools. These included the proportion of visits conducted virtually before and during the pandemic and the expected volume post-pandemic. They also assessed the overall experience with offering virtual visits, the different platforms and methods used when offering virtual visits, and the comfort level with these methods. Other questions explored participants’ opinions of the barriers to adopting virtual visits and the perception of useful resources and support tools. Additional survey questions addressed EMR use and experience and level of comfort using technology in general. The survey also collected providers’ demographic and practice information, including age, years of practice in Canada, gender, practice settings size, and practice geography (urban or rural).

In general, the questionnaire used a Likert scale (five-point and three-point scales) and a multiple-choice format, where respondents were asked to choose all options that applied for some of the questions.

Respondents were anonymous, with no way to link responses to responders. Participation in this study was entirely voluntary. Participants had the choice to decline any of the survey questions they did not wish to answer.

This descriptive cross-sectional survey study had ethics approval from the Hamilton Integrated Research Ethics Board (HIREB) under Project 11389.

## Statistical analysis

Study data were managed and analyzed using the Statistical Package for Social Sciences (SPSS) (SPSS, IBM Corp, Armonk, New York. Version 27; 2020). Descriptive statistics of survey responses were calculated, and summary data was displayed as frequencies (%) or mean.

Bivariate analyses were conducted. Chi-square tests and Fisher’s Exact tests were used to examine the relationship between EMR and non-EMR integrated virtual visits’ platforms, challenges, useful resources and support tools, and the virtual visit method. The tests were also used to assess the relationship between participants’ characteristics and their perception of challenges, useful resources and helpful support tools. Where applicable, post hoc multiple z-tests of two proportions or multiple Fisher’s exact tests followed up statistically significant tests, both with a Bonferroni correction to adjust for multiple comparisons.

We examined the satisfaction and comfort level comparisons using a ranked-based nonparametric Kruskal-Wallis H test. A one-way analysis of variance (ANOVA) test was conducted to determine if a difference exists between the mean proportion of virtual visits offered in response to COVID-19 in relation to the different modalities used (phone calls, video, secure messages and text messages) for the virtual visits. The multiple comparison test using Tukey adjusted p values was used to identify significant associations between groups.

Chi-square test with the Yates’ continuity correction was used to examine for any statistically significant differences in the use of secure messaging in relation to the type of platform used (remunerated vs. non-remunerated).

A 2-sided P-value <0.05 was considered statistically significant. Original P values are shown in the results tables, and footnotes indicate whether the P levels were <0.0125 after the adjustment.

## Results

### Demographic characteristics

Overall, 207 family physicians and nurse practitioners responded to the survey. Of those who responded, 200 offered virtual visits in their practice (96.6%). The majority (80.5%) were family physicians. Slightly greater than half of the respondents (60.6%) were females, 71% had an urban practice, and 44% worked at a medium-sized group practice setting. About half of respondents (54.2%) had been practising in Canada for 15 years and over. As summarized in Table 1, 60.7% of respondents had more than five years of experience with EMR use; about 96.1% indicated an average to an expert level in using EMR and technology in general (Table 1). In terms of the Ontario Health Teams, the responses originated mainly from Kitchener, Waterloo, Wellesley, Wilmot, and Woolwich (KW4), Hamilton, and Niagara OHTs (16.7%, 15.6%, and 15.0%, respectively) (S1 Fig).

**Table 1:** Characteristics of respondents.

Characteristics	All Participants
	N (%)
**Role**	
Family physician (FP)	163 (80.5%)
Nurse practitioners (NP)	37 (19.5%)
**Total**	200
**Sex**	
Male	69 (39.4%)
Female	106 (60.6%)
**Total**	175
**Years of Practice**	
≤15 y	82 (45.8%)
>15 y	97 (54.2%)
**Total**	179
**Practice Geography**	
Urban	125 (70.6%)
Rural	52 (29.4%)
**Total**	177
**EMR use**	
≤5 y	70 (39.3%)
>5 y	108 (60.7%)
**Total**	178
**Practice size**	
Solo	30 (17.1%)
Small group	33 (18.9%)
Medium group	77 (44.0%)
Large group	35 (20.0%)
**Total**	175
**Comfort level with EMR**	
Novice	7 (3.9%)
Average	97 (54.5%)
Expert	74 (41.6%)
**Total**	178
**Comfort level with technology**	
Novice	6 (3.2%)
Average	117 (62.9%)
Expert	63 (33.9%)
**Total**	186

* Total responses are different as participants had the option to decline responding to any of the questions.

### Clinicians’ experience with virtual visit use

#### Overview of virtual visit use by method

A total of 200 participants offered virtual visits in their practice settings. Participants used different modalities to offer virtual visits, with the vast majority in the form of phone calls (N = 199; 99.5%), 67.0% (N = 134) offered video calls, 41.5% (N = 83) used secure messages, and 18.5% (N = 37) used text messaging.

#### Subgroup analyses

*Differences in responses based on years of practice*. Clinicians who had more than 15 years of practice experience were significantly more likely to use text messaging to connect with patients than those with 15 or fewer years of practice experience (24.7% vs. 11.0%, P = 0.021) (Table 2).

**Table 2:** Methods and platform use for virtual visits by respondents characteristics.

	Participants use phone calls N = 199 (99.5%) N (%)	Participants use video visits N = 134 (%) N (%)	Participants use secure messaging N = 83 (%) N (%)	Participants use text N = 37 (%) N (%)	EMR integrated N = 92 (45%) N (%)	Non- EMR integrated N = 152 (76%) N (%)
**Role**						
FP	162 (99.4%)	105 (64.4%)	73 (44.8%)	33 (20.2%)	78 (47.9%)	120 (73.6%)
NP	37 (100%)	29 (78.4%)	10 (27.0%)	4 (10.8%)	14 (37.8%)	32 (86.5%)
***P value***	*0*.*851*	*0*.*073*	***0*.*035***	*0*.*134*	0.496	0.098
**Sex**						
*Male*	68 (100.0%)	44 (64.7%)	29 (42.6%)	15 (22.1%)	35 (51.5%)	52 (76.5%)
*Female*	106 (100.0%)	73 (70.2%)	46 (44.2%)	16 (15.4%)	52 (50.5%)	80 (76.9%)
***P value***		*0*.*451*	*0*.*876*	*0*.*266*	*0*.*877*	*0*.*945*
**Years of Practice**						
≤15 y	82 (100.0%)	53 (64.6%)	35 (42.7%)	9 (11.0%)	36 (43.9%)	67(81.7%)
>15 y	97 (100.0%)	67 (69.1%)	41 (42.3%)	24 (24.7%)	54 (55.7%)	70 (72.2%)
***P value***		*0*.*623*	*0*.*955*	***0*.*021***	*0*.*135*	*0*.*133*
**Practice Geography**						
Urban	125(100.0%)	85(66.9%)	56(44.1%)	25(19.7%)	70 (55.1%)	94 (74.0%)
Rural	51(100.0%)	34 (66.7%)	20 (39.2%)	7 (13.7%)	20 (39.2%)	42 (82.4%)
***P value***		*0*.*973*	*0*.*617*	*0*.*396*	*0*.*065*	*0*.*236*
**EMR use**						
≤5 y	69 (100.0%)	47(68.1%)	19(27.5%)	10(14.5%)	37(53.6%)	56(81.2%)
>5 y	105(100.0%)	70(66.7%)	56(53.3%)	18(17.1%)	52(49.5%)	77(73.3%)
***P value***		*0*.*870*	***0*.*001***	*0*.*679*	*0*.*597*	*0*.*234*
**Practice size**						
Solo	28 (100.0%)	14 (50.0%)	11 (39.3%)	5 (17.9%)	12 (42.9%)	18 (64.3%)
Small group	32 (100.0%)	18(56.3%)	11(34.4%)	9(28.1%)	17(53.1%)	23(71.9%)
Medium	76 (100.0%)	54 (71.1%)	30 (39.5%)	14 (18.4%)	36 (47.4%)	60 (78.9%)
Large group	35 (100.0%)	27 (77.1%)	21 (60.0%)	4 (11.4%)	22 (62.9%)	29 (82.9%)
***P value***		*0*.*061*	*0*.*130*	*0*.*376*	*0*.*366*	*0*.*299*
**Comfort level with EMR**						
Novice	7 (100.0%)	2 ^b^ (28.6%)	2 (28.6%)	2 (28.6%)	3 (42.9%)	6 (85.7%)
Average	97 (100.0%)	61 ^a^ (64.9%)	35 (37.2%)	16 (17.0%)	44 (46.8%)	71 (75.5%)
Expert	73 (100.0%)	55 ^a^ (75.3%)	39 (53.4%)	13 (17.8%)	43 (58.9%)	55(75.3%)
***P value***		***0*.*027***	*0*.*080*	*0*.*739*	*0*.*268*	*0*.*824*
**Comfort level with technology**						
Novice	6 (100.0%)	3 (50.0%)	2 (33.3%)	3 (50.0%)	2 ^a^ (33.3%)	6 (100.0%)
Average	115 (100.0%)	72 (62.1%)	43 (37.1%)	22 (19.0%)	50 ^b^ (43.1%)	86 (74.1%)
Expert	63 (100.0%)	47 (78.3%)	32 (53.3%)	8 (13.3%)	38 ^b^ (63.3%)	48 (80.0%)
***P value***		*0*.*062*	*0*.*106*	*0*.*078*	***0*.*028***	*0*.*269*

*Significant difference using Post hoc analysis using Bonferroni correction was accepted at *p* <0.0125. Different letters between groups = Significant difference; same letters between group = Non-significant difference.

*Differences in methods use to provide virtual care based on clinicians’ role*. Family physicians were significantly more likely to use secure messaging in providing virtual care than nurse practitioners (44.8% vs. 27.0%, P = 0.035) (Table 2).

*Differences in responses based on EMR use and comfort level*. In our statistical analysis, subgroups were created for clinicians with ≤5 years vs. >5 years of EMR use. Clinicians more experienced with EMR use were more likely to use secure messaging as a virtual communication method than less experienced EMR users (53.3% vs. 27.5%, P = 0.001) (Table 2).

Clinicians who were more comfortable with EMR or technology (average or expert levels) were more likely to conduct video visits or use an integrated platform with their EMR to offer virtual visits than novice EMR and technology users (Table 2).

No other significant differences were found for the different modalities and platforms used to offer the visits virtually in relation to the clinicians’ characteristics (Table 2).

#### Virtual visit use in response to COVID-19 pandemic

Since the COVID-19 pandemic, clinicians who offered virtual visits have conducted an average of 66.4% of their visits virtually, compared to an average of 6.5% before COVID-19. Participants anticipated continuing the use of virtual communication to conduct an average of 43.9% of their visits post-pandemic.

No significant differences were detected between the proportion of virtual visits offered/anticipated to be provided pre-, during, and post-pandemic in relation to the different modalities used to offer visits virtually.

#### Overview of virtual use by platform

Participants used different platforms to conduct virtual visits. About three-quarters of participants (76%; 152/200) used non-EMR integrated platforms to offer virtual visits. Almost half of the participants (45%; 92/200) also used integrated platforms with their EMR. S2 Fig summarizes the adoption of different platforms—EMR integrated and non-integrated—among participants.

No significant differences were detected between the different virtual visit modalities used in relation to the platform type (EMR integrated vs. non-integrated).

However, clinicians were significantly more likely (P = 0.012) to use secure messaging to connect virtually with patients using platforms that didn’t include remuneration for those visits (83.1%; 69/83) compared to the use of secure messaging from a platform where remuneration was available (Novari and Think Research) (18.0%; 15/83).

#### Level of satisfaction with virtual visits

Most of the participants (92.4%) indicated they were comfortable/very comfortable with the use of phone calls, 62% with video visits, 74.6% with the use of secure messaging, and 75% were comfortable/very comfortable using text messages (Table 3). Overall, 74.5% of participants were satisfied/very satisfied with their experience using virtual visits. The majority (88%) believed they could incorporate virtual visits well within the usual workflow.

**Table 3:** Satisfaction and comfort level with different modalities of virtual visits.

	All participants N = 200 N (%)	Participants use phone calls N = 199 (99.5%) N (%)	Participants use video visits N = 134 (67.0%) N (%)	Participants use secure messaging N = 83 (41.5%) N (%)	Participants use text N = 37 (18.5%) N (%)	P value
**Overall satisfaction**						
*Very satisfied*	58 (29.0%)	57 (28.7%)	44 (32.8%)	26 (31.3%)	12(32.4%)	
*Satisfied*	91 (45.5%)	91 (45.8%)	65 (48.6%)	41 (49.4%)	17 (45.9%)	0.548
*Neutral*	36 (18.0%)	35 (17.9%)	15 (11.2%)	13 (15.7%)	6 (16.3%)	
*Dissatisfied*	12 (6.0%)	12 (6.1%)	8 (6.0%)	3 (3.6%)	2 (5.4%)	
*Very Dissatisfied*	3 (1.5%)	3 (1.5%)	2 (1.4%)	0 (0.0%)	0 (0.0%)	
**Comfort level using different modes**						
*Very comfortable*		131(65.8%)	45 (22.5%)	32 (38.5%)	11 (29.7%)	0.615
*Comfortable*		53(26.6%)	79 (39.5%)	30 (36.1%)	16 (43.3%)	
*Neutral*		10 (5.0%)	4 (17.0%)	12 (14.4%)	7 (18.9%)	
*Uncomfortable*		2 (1.0%)	2 (6.5%)	7 (8.4%)	2 (5.4%)	
*Very Uncomfortable*		3 (1.6%)	4 (3.4%)	3 (3.5%)	1 (2.7%)	
**Integrate well with daily workflow**						
Yes	176 (88.0%)	175 (87.9%)	121 (90.2%)	73 (87.9%)	30 (81.0%)	0.336

No significant differences were detected between participants in relation to the modalities used to offer virtual visits (Table 3).

#### Subgroup analyses

*Differences in satisfaction between male and female participants*. Females were significantly more satisfied than males with offering virtual visits to connect with patients (66.7% vs. 33.3%, P = 0.006).

*Differences in responses based on years of practice*. Clinicians with fewer years of experience (≤15 years) were significantly more satisfied with virtual visit digital tools compared to those with >15 years of practice (82.9% vs. 68.0%, P = 0.025).

No other significant differences were detected in satisfaction levels between participants in relation to their characteristics.

### Challenges, useful support tools, and resources to offering virtual visits

#### Challenges to offering virtual visits

Participants highlighted some challenges in offering virtual care. For example, 58% (116/200) of participants were concerned about patients’ limited access to technology and devices and 55% (110/200) about their degree of technology knowledge. Around 41% (82/200) of clinicians thought that the lack of integration with their current EMR, along with the increase in demand for time (41%; 82/200) and connectivity issues such as inconsistent Wi-Fi/Internet connection (41%; 82/200), are challenges to providing virtual visits.

Also, 32% (64/200) of clinicians were worried about the limited resources—such as adequate administrative support and technology, and 33% (66/200) were concerned with patients overusing the virtual service. Concerns about patient privacy and obtaining email consent at 28% (56/200) and 16.5% (33/200), respectively, were among the challenges to offering virtual visits. Only 10.5% (21/200) and 9.0% (18/200) of clinicians thought that inability to justify the cost of virtual visits and the lack of adequate training poses a challenge to use virtual visits in practice.

No statistically significant differences in challenges were detected in relation to the different modalities used to offer virtual visits.

#### Subgroup analyses

*Differences in perspectives and use between male and female participants*. Females were significantly more likely than males to consider insufficient administrative support to delegate tasks (38.5% vs. 23.5%, P = 0.032) as a challenge to incorporating virtual visits within their practice. Also, females were more likely than males to believe that patients’ limited access to technology (71.2% vs. 52.9%, P = 0.023) can be a barrier. On the other hand, males have significantly considered the ability to justify the cost of virtual visits as a barrier to integrating the virtual tool compared to females (19.1% vs. 5.8%, P = 0.007) (Table 4).

**Table 4:** Challenges to conducting virtual visits by the characteristics of respondents.

	Unable to justify cost	Concerns increase demand	Adequate Training	Adequate admin support	Limited technology knowledge	Connectivity issues	Patients overusing services	Patient access to technology
	N = 21 (10.5%)	N = 82 (41.0%)	N = 18 (9.0%)	N = 64 (32.0%)	N = 110 (55.0%)	N = 82(41.0%)	N = 66 (33.0%)	N = 116 (58.0%)
	N (%)	N (%)	N (%)	N (%)	N (%)	N (%)	N (%)	N (%)
**Role**								
FP	20 (12.9%)	74 (45.4%)	13 (8.0%)	55 (33.7%)	69 (42.3%)	66 (40.5%)	60 (36.8%)	88 (54.0%)
NP	1 (2.7%)	8 (21.6%)	5 (13.5%)	9 (24.3%)	18 (48.6%)	15 (40.5%)	6 (16.2%)	28 (75.7%)
***P value***	***0*.*021***	***0*.*006***	*0*.*288*	0.331	0.484		***0*.*011***	***0*.*016***
**Sex**								
Male	13 (19.1%)	29 (42.6%)	3(4.4%)	16 (23.5%)	28 (41.2%)	30 (44.1%)	20(29.4%)	36 (52.9%)
Females	6 (5.8%)	42 (40.4%)	12(11.5%)	40 (38.5%)	52(50.0%)	51(49.0%)	39 (37.5%)	74(71.2%)
***P value***	***0*.*007***	*0*.*874*	*0*.*087*	***0*.*032***	*0*.*277*	*0*.*537*	0.325	***0*.*023***
**Years of Practice**								
≤15 y	12(14.6%)	31(37.8%)	4(4.9%)	25(30.5%)	38(46.3%)	36(43.9%)	33(40.2%)	51(62.2%)
>15 y	8 (8.2%)	43(44.3%)	13(13.4%)	32(33.0%)	44(45.4%)	46(47.4%)	28(28.9%)	60(61.9%)
***P value***	*0*.*234*	0.447	**0.*044***	*0*.*750*	*0*.*896*	*0*.*654*	*0*.*117*	*0*.*963*
**Practice Geo**								
Urban	16 (12.6%)	57(44.9%)	14(11.0%)	41(32.3%)	51(40.2%)	51(40.2%)	50(39.4%)	74(58.3%)
Rural	4 (7.8%)	17(33.3%)	3(5.9%)	15(29.4%)	30(58.8%)	30 (58.8%)	11(21.6%)	36(70.6%)
***P value***	*0*.*441*	*0*.*181*	0.402	0.858	***0*.*030***	***0*.*030***	***0*.*024***	***0*.*042***
**EMR use**								
≤5 y	5(7.2%)	21(30.4%)	6(8.7%)	35(36.2%)	37(53.6%)	35(50.7%)	21(30.4%)	51(73.9%)
>5 y	13(12.4%)	50 (47.6%)	11(10.5%)	30(28.6%)	44(41.9%)	46(43.8%)	39(37.1%)	58(55.2%)
***P value***	*0*.*319*	***0*.*028***	*0*.*798*	*0*.*320*	*0*.*162*	*0*.*438*	*0*.*416*	***0*.*016***

*Significant difference using Post hoc analysis using Bonferroni correction was accepted at *p* <0.0125. Different letters between groups = Significant difference; same letters between group = Non-significant difference.

**Participants were invited to choose all that apply for the questions.

***Participants had the option to decline responding to any of the questions.

*Differences in responses between family physicians and nurse practitioners*. Family physicians were significantly more likely than nurse practitioners to consider the increase in demand for virtual care, patients overusing the services and the ability to justify the cost of virtual visits as challenges to incorporating virtual visits within their practice (45.4% vs. 21.6%, P = 0.006; 36.8% vs. 16.2%, P = 0.011; and 12.9% vs. 2.7%, P = 0.021, respectively). Conversely, nurse practitioners were more likely to believe that patients’ limited access to technology (75.7% vs. 54.0%, P = 0.016) can be a barrier (Table 4).

*Differences in responses based on years of practice*. Clinicians with more than 15 years of practice were significantly more likely to consider the lack of adequate training and education of the digital tools for virtual care as a challenge to conducting virtual visits compared to those with less than 15 years of experience (13.4% vs. 4.9%, P = 0.044) (Table 4).

*Differences in responses based on practice geography*. More than half of clinicians whose practice was in a rural area were more likely to consider the inconsistent Wi-Fi and limited Internet connection as barriers to integrating virtual visits within the practice setting than those practising in urban settings (58.8% vs. 40.2%, P = 0.030). Clinicians whose practice was in a rural area were also more likely than those practising in urban areas to consider patients’ limited access to technology (70.6% vs. 58.3%, P = 0.042) and patients’ limited knowledge of technology (58.8% vs. 40.2%, P = 0.030) as an added challenge. However, clinicians whose practice was in an urban setting were significantly more concerned than those in rural settings about patients overusing virtual care services (39.4% vs. 21.6%, P = 0.024) (Table 4).

*Differences in responses based on EMR use*. Compared to clinicians with less EMR experience, those with more experience with EMR use were more likely to be concerned about patients’ increased demand for virtual care (47.6% vs. 30.4%, P = 0.028) and less likely to worry about patients’ access to technology (55.2% vs. 73.9%, P = 0.016) (Table 4).

No significant differences were detected in participants’ opinions of other challenges to offer virtual visits in relation to their characteristics.

#### Useful support tools, and resources to offering virtual visits

When asked about useful supports for integrating virtual visits in practice, 47% (94/200) of clinicians indicated that standards for virtual care outlined by their profession’s college would be a helpful support resource. Also, 29.5% (59/200) of clinicians highlighted the usefulness of accessible resources on virtual care platforms. In comparison, 16.5% (33/200) and 24.0% (48/200) supported written information and the offering of evidence on virtual care effectiveness, respectively.

Additionally, 32.5% (65/200) of clinicians identified change management supports, such as support with workflow integration and defining roles in the team, and 32.0% (64/200) recognized technical training on using virtual care tools as efficient support processes. Also, 39% (78/200) and 28% (56/200) of respondents reported that the local colleague support and in-house organizational support are helpful resources.

#### Subgroup analyses

*Differences in the opinion of support tools between male and female participants*. Females were significantly more likely to consider local colleague support as a useful support tool than males (49.0% vs. 23.5% P = 0.001). Females were also more likely to recognize the use of available resources to compare virtual visit platforms (cost, features, pros/cons, etc.) (38.5% vs. 23.5%, P = 0.047) as a useful resource for incorporating virtual visits within their practice setting (Table 5).

**Table 5:** Useful support tools and resources to offereing virtual visits by the characteristics of respondents.

	Local colleague support	In-house organizational supports	Change management supports	Technical training on how to use tool	Connection with a colleague	Written information	Evidence on effectiveness	Resources on platforms
	N = 78 (39.0%)	N = 56 (28.0%)	N = 65 (32.5%)	N = 64(32.0%)	N = 33(16.5%)	N = 33(16.5%)	N = 48 (24.0%)	N = 59(29.5%)
	N (%)	N (%)	N (%)	N (%)	N (%)	N (%)	N (%)	N (%)
**Role**								
FP	55 (33.7%)	41 (25.2%)	57 (35.0%)	53 (32.5%)	27 (16.6%)	30 (18.4%)	36 (22.1%)	47 (28.8%)
NP	23 (62.2%)	15 (40.5%)	8 (21.6%)	11 (29.7%)	6 (16.2%)	3 (8.1%)	12 (32.4%)	12 (32.4%)
***P value***	***0*.*001***	0.060	0.118	0.846	0.959	0.128	0.183	0.665
**Sex**								
Male	16 (23.5%)	17 (25.0%)	19 (27.9%)	18 (26.5%)	13(19.1%)	11(16.2%)	15 (22.1%)	16 (23.5%)
Females	51 (49.0%)	29 (27.9%)	37 (35.6%)	41 (39.4%)	17(16.3%)	17 (16.3%)	27(26.0%)	40 (38.5%)
***P value***	***0*.*001***	*0*.*727*	*0*.*322*	*0*.*101*	*0*.*684*	0.976	*0*.*591*	***0*.*047***
**Years of Practice**								
≤15 y	31 (37.8%)	19 (23.2%)	31 (37.8%)	22(26.8%)	14(17.1%)	14 (17.1%)	19 (23.2%)	29 (35.4%)
>15 y	40 (41.2%)	31 (32.0%)	27 (27.8%)	38 (39.2%)	16(16.5%)	14 (14.4%)	25 (25.8%)	27 (27.8%)
***P value***	*0*.*649*	*0*.*242*	*0*.*200*	*0*.*112*	0.*918*	*0*.*682*	*0*.*730*	*0*.*332*
**Practice Geography**								
Urban	46 (36.2%)	36 (28.3%)	41 (32.3%)	41 (32.3%)	21 (16.5%)	20 (15.7%)	33 (26.0%)	39(30.7%)
Rural	25 (49.0%)	14 (27.5%)	17 (33.3%)	19 (37.3%)	9 (17.6%)	8 (15.7%)	11 (21.6%)	17(33.3%)
***P value***	*0*.*130*	*0*.*904*	*0*.*893*	*0*.*599*	0.828	*0*.*992*	*0*.*572*	*0*.*725*
**EMR use**								
≤5 y	29 (42.0%)	22 (31.9%)	24 (34.8%)	20 (29.0%)	11 (15.9%)	10 (14.5%)	20 (29.0%)	22 (31.9%)
>5 y	42 (40.0%)	26 (24.8%)	33 (31.4%)	38 (36.2%)	19 (18.1%)	18 (17.1%)	24 (22.9%)	34 (32.4%)
***P value***	*0*.*875*	*0*.*386*	*0*.*741*	*0*.*411*	*0*.*838*	*0*.*679*	*0*.*378*	*0*.*945*
**Practice size**								
Solo	6 (21.4%)	6 (21.4%)	9 (32.1%)	11(39.3%)	6 (21.4%)	6 (21.4%)	6 (21.4%)	7 (25.0%)
Small group	15 (46.9%)	11 (34.4%)	11 (34.4%)	15 (46.9%)	7 (21.9%)	7 (21.9%)	11 (34.4%)	11 (34.4%)
Medium group	32 (42.1%)	22 (28.9%)	23 (30.3%)	22 (28.9%)	8 (10.5%)	10 (13.2%)	13 (17.1%)	27 (35.5%)
Large group	14 (40.0%)	11 (31.4%)	13 (37.1%)	8 (22.9%)	7 (20.0%)	3 (8.6%)	11 (31.4%)	8 (22.9%)
***P value***	*0*.*189*	*0*.*725*	*0*.*905*	0.139	0.326	0.326	0.169	0.484
**Comfort level with EMR**								
Novice	5^a^ (71.4%)	1(14.3%)	3(42.9%)	3 (42.9%)	2 (28.6%)	2 (28.6%)	4 (57.1%)	3(42.9%)
Average	43^a^ (45.7%)	23(24.5%)	30 (31.9%)	38 (40.4%)	15 (16.0%)	15 (16.0%)	24 (25.5%)	39 (30.9%)
Expert	22^b^ (30.1%)	24(32.9%)	25 (34.2%)	17 (23.3%)	12 (15.1%)	11 (15.1%)	16 (21.9%)	23 (31.5%)
***P value***	***0*.*029***	*0*.*350*	*0*.*819*	*0*.*067*	*0*.*687*	*0*.*648*	*0*.*122*	*0*.*805*

*Significant difference using Post hoc analysis using Bonferroni correction was accepted at *p* <0.01. Different letters between groups = Significant difference; same letters between group = Non-significant difference.

**Participants were invited to choose all that apply for the questions.

***Participants had the option to decline responding to any of the questions.

*Differences in responses between family physicians and nurse practitioners*. Nurse practitioners were significantly more likely to recognize the local support as a helpful support resource to incorporating virtual care in practice (62.2% vs. 33.7%, P = 0.001) (Table 5).

*Differences in the opinion of support tools based on the level of EMR experience*. Clinicians who rated their comfort level with their current EMR as novice users were more likely to consider local colleague support as a helpful support tool for incorporating virtual care into their workflow (P = 0.029) (Table 5).

## Discussion

The study’s main objective was to assess primary care practitioners’ use of virtual visits in primary care settings across Southwestern Ontario after the first wave of the COVID-19 pandemic. Our results showed that the use of virtual care has increased in primary care settings with the COVID-19 pandemic. The average rate of using virtual visits has increased by 59.9%, with anticipation to maintain an average of 43.9% of clinical visits virtually post-pandemic. About 83% of primary care clinicians used synchronous methods with a 92% average comfort level for offering these modalities, and 30% used asynchronous methods with an average comfort level of 74% for providing virtual visits. Clinicians also used both EMR (45%) and non-EMR (76%) integrated platforms. Overall, this descriptive study sheds light on tools and resources that could enhance operational efficiencies to adopting virtual visits in primary care settings and highlights challenges that, when addressed, can expand the health system capacity and sustained use of virtual care.

### Primary care clinicians’ experience with virtual care

Since the pandemic, the Ontario healthcare system endorsed several different virtual modes within the practice settings to clinically interact with patients [13]. Participants in our study reported high satisfaction with offering virtual care (74.5%) and moderate to high comfort levels with the different virtual visit modalities (92.4% comfortable with phone calls, 62% with video visits, 74.6% with secure messaging, and 75% with text messages). No significant differences were noted between the comfort levels in using the different modalities of virtual tools. Our findings align with Dixon and Stahl’s results from a pilot study where clinicians were satisfied with the quality of the different modalities used to conduct virtual visits [23]. Moreover, they reported that patients felt that clinicians were moderately comfortable with conducting video visits [23].

Females were significantly more satisfied than males in communicating with patients virtually (66.7% vs. 33.3%, P = 0.006). Evidence shows that female PCPs tend to spend an average of two minutes more per patient than males [24]. The flexible schedule with virtual care could alleviate the burden on them and maintain their productivity, hence increasing their satisfaction with the virtual communication methods.

User-friendly digital tools play an essential role in determining the future mode of delivery of virtual care. The ease, satisfaction, and moderate comfort in using the various existing virtual care modalities reported in this study might have facilitated the 59.9% sharp rise in adoption upon the emergence of the pandemic. Our findings are consistent with a recently published study that reported a 56-fold increase in virtual visits’ uptake during the pandemic compared to the pre-COVID-19 period [25]. The reported dramatic shift in primary care providers’ use of virtual care during the pandemic exhibits primary care’s resiliency in utilizing various virtual modalities and demonstrates a capacity to adopt simple digital tools quickly.

In our study, clinicians used synchronous methods to offer virtual visits (99.5% used phone calls and 67.0% used video calls) more than asynchronous modes (41.5% used secure messages and 18.5% used text messaging). This variability could be related to the clinicians’ reported concerns about privacy, which could be more of an issue with stored messages, the somewhat similar nature of synchronous methods to the typical office visit, and to some extent, the differences in the patients’ conditions. Interestingly, when the asynchronous methods were used, text messages were significantly more utilized among clinicians who had been in practice for more than 15 years (24.7% vs.11.0%, P = 0.021). This could be due to a long-term relationship with patients or having patients with mild conditions or manageable follow-up where texts are the simplest and quickest mode to use. When Dixon and colleagues in their study compared the asynchronous visits to other modalities of visits used to follow up with patients with high blood pressure, they did not find any significant differences in outcome. However, the asynchronous visits were favoured because of the 20% in time savings conducting or documenting the visit [26].

Our respondents used a heterogenous mix of virtual visit platforms, which were both EMR and non-EMR integrated. With the sudden need to adopt virtual care in practice, non-integrated platforms seemed to have had an advantage, with clinicians using 31% more of these platforms compared to those that are EMR integrated. This may highlight the value of the simplicity of technology to support enhanced adoption of virtual technology. The level of comfort noted among participants in this study is encouraging as a baseline measure, highlighting the potential for further sustainment of the use of virtual care options post-pandemic.

### Challenges to the current model of virtual care

The geographic location where primary care providers practice, either rural or urban, may challenge the capacity to provide virtual care. In our study, clinicians practicing in rural areas were significantly more concerned about their patients’ limited access to technology (70.6% vs. 58.3%, P = 0.042) and inconsistent Wi-Fi and connectivity issues (58.8% vs. 40.2%, P = 0.030) than those practicing in urban locations and thought of these factors as barriers to access virtual care.

Several platforms and modalities used to offer virtual care require the internet to operate appropriately. The Canadian Radiotelevision and Telecommunications Commission found that 87.4% of Canadian households have access to download speeds of at least 50 megabits per second and upload ten megabits per second. Conversely, in rural communities, just 45.7% of households have equal access [27]. Glazier and colleagues reported that rural residents were among those with the lowest virtual care use at 60.6% during the pandemic [25]. Another review conducted by Li and colleagues in 2020 underscored the need to address virtual care’s technical capacity in rural communities [28]. Our findings showed that 58% of respondents had concerns about patients’ ability to adequately access technological tools. In some regions, limited connectivity might pose limitations to the type of virtual visit offered to interact with patients. When equitable access to reliable broadband internet speeds is not always available, video visits may not be feasible. Thus, phone calls and secure messaging may be the main methods of virtual communication [29]. If this issue is not tackled, this risk would negatively contribute to the current digital divide and inequitable access to virtual care in Canada [25].

In contrast to rural settings, many primary care providers in urban locations and their patients benefit from access to acceptable bandwidth required to engage in a virtual environment. Therefore, clinicians in urban areas posed different concerns around overutilization and fears of increasing virtual visit demands. Respondents in urban settings were more concerned about patients overutilizing services than their colleagues in rural locations (39.4% vs. 21.6%, P = 0.024). However, other research has shown that patients have been respectful of their providers’ time and have typically used virtual care appropriately, specifically in regards to the use of secure messaging [6, 8]. To support appropriate access to virtual care, it is essential to provide a universal list of approved medical conditions for virtual care covered within the government-funded health care boundaries [29]. This needs to happen through a broader implementation initiative. Engaging all relevant stakeholders—policymakers, clinicians, and patients—can facilitate the adequate adoption of technology that best meets everyone’s needs and ensures quality care [30].

The sudden need to adopt digital technologies in practice settings to address access to care during the COVID-19 pandemic forced clinicians to use technologies they feel more comfortable using, whether secure or insecure. Interestingly, only 28% of our study respondents were concerned about patient privacy in a virtual platform. However, privacy must remain a priority as virtual care continues. Some technological devices and applications may not fall under the current privacy protection policies [31]. Privacy and security standards for digital technologies are essential for defining the quality of virtual care delivery.

Clinicians were more likely to use secure messaging, when available, regardless of the platform. This finding aligns with Stamenova and colleagues who reported that both patients and clinicians in rostering settings favour secure messaging [6]. In our study, remuneration was not a factor in pursuing secure messaging. Instead, clinicians used it even more with platforms that did not offer it for those visits. This is, perhaps, due to the ambiguity of the fee for service policies with the current contract agreements, salaried physicians and session fees plans for the remunerated platforms [32].

Therfore, usability and interoperability of the system used are other important factors to consider [8, 33]. In our study, 41% of clinicians were concerned about the lack of integration with their current EMR. Many of the heterogeneous platforms currently available within the healthcare system have limited integration with the EMR. Adding more digital solutions with poor interoperability with the current system complicates virtual visits incorporation within the clinical workflow. The variety of the available platforms is likely a reflection of Ontario’s diverse number of certified electronic medical record systems and the open procurement rules in primary care [16]. Clinicians in our study indicated an interest to continue the use of virtual visits with an average of 43.9% post-pandemic. To ensure the sustainability of virtual visits’ service in the primary care setting, comprehensive models with a standardized paradigm incorporating usability and interoperability elements are needed. This will ensure comfort with the use of different platforms and seamless integration with existing workflows.

### Support tools for primary care

Clinicians indicated that they would benefit from resources to integrate virtual care into practice. Our study showed that female PCPs are more likely than their male counterparts to utilize the available resources to incorporate virtual visits in practice. Furthermore, almost half of the clinicians(47%), in general, highlighted the standards for virtual care developed by their professional College as essential support. The College of Nurses of Ontario updated their guidelines for Telepractice in 2020 [34]. The College of Family Physicians of Canada, the Canadian Medical Association, and the Royal College of Physicians and Surgeons of Canada published the Virtual Care Playbook in 2020 [20]. However, not all colleges have produced or updated standards for virtual care. Evidence-based standardized guidelines from each professional College would be a useful resource to support clinicians with virtual technology adoption.

Interestingly, 39% of clinicians identified that a connection to a local peer who uses the technology seemed to be of added value to incorporate virtual care into practice. This was especially true for novice EMR users and female physicians. This is important in future digital planning within primary care settings. Records on female physicians’ attitudes at workplaces and in practice are understudied. Our study showed that female PCPs are more likely than their male counterparts to reach out to a local colleague for support to incorporate virtual visits in practice. These findings align with McMurray and colleagues who reported significantly greater experience among female physicians with approachable physician colleagues at the workplace while managing care [35]. A group of local clinicians who can support others with virtual visit adoption would be an asset to the technology’s sustainable uptake. This finding is consistent with the diffusion of innovation theory. There are different elements to the diffusion of innovation theory that allow for a new idea to spread and diffuse among potential users. One of the aspects to adopting the latest digital technology would be the compatibility of the novel method or technique to users’ current practices and needs [36]. Another critical element for the initial and continued use of the innovation is observing other adopters through the peer network [36]. This would allow for informal interaction and the creation of common ground that alleviates readiness resistance to adopting the technology [37].

Having change management support, technical training, in-house organizational and administrative support were also recognized as valuable local resources to support the integration of virtual visits in primary care settings. Evidence shows that a change management framework is considered an asset to introducing and sustaining innovative technology [38, 39]. The primary care sector comprises clinics that traditionally act as independent operating organizations [40, 41]. Therefore, it is essential to prepare a comprehensive, collaborative support model that includes adequate organizational resources, evidence-based information, a robust local change management plan, and regional champions to support virtual care adoption and sustained use in workflows.

### Limitations

Although this study obtained a fair representation of clinicians from different Ontario Health Teams across Southwestern Ontario, the study was limited by the variability of the distribution of responses from Ontario Health Teams and the potential self-selection bias among participants. Also, patterns of use and experience among clinicians in the southwestern region might differ from the other regions, limiting our findings’ generalizability to all primary care practice settings at the provincial level.

This descriptive cross-sectional survey considered only community primary care practitioners’ perspectives on their experience with virtual visits. Future studies in which the specialists’ views are collected would give a broader vision of the virtual visits within the local healthcare system.

Our survey responses were also collected at a point in time after the first wave of the pandemic in Ontario, which may not accurately reflect the clinicians’ experience with virtual visits over time and with different circumstances and settings. Further prospective exploration of clinicians’ perspectives of virtual visits is necessary.

Despite these limitations, it is worth noting that our study explored the perspectives of clinicians’, in rural and urban areas, at a distinctive time of a pandemic and with the use of various modalities of virtual care. This study provides evidence of clinicians’ current experience with the available support tools and challenges to adopting virtual visits in a primary care setting during the pandemic. Our findings have substantial implications that could support efforts currently underway by governments and regulatory bodies to determine how to sustain virtual access to care in Canada’s healthcare system.

Our findings highlight the importance of equitable cellular access and connectivity among Canada’s different geographic locations to ensure quality patient-provider interactions. Additionally, a comprehensive supportive paradigm based explicitly on local resources, technical, administrative, change management, and local clinicians’ support would ensure a seamless adoption of new technology like virtual visits in primary care.

## Conclusions

Many primary care practitioners have moved towards incorporating virtual care into their practice. Our study shows that the adoption of virtual visits has dramatically increased during the pandemic, and clinicians have experienced a high satisfaction level with the technology. There is also substantial interest in continuing to use virtual care among primary care practitioners post-pandemic. Adequate local resources and evidence-based information would help clinicians continue with the use of virtual care. However, our findings highlight the need for further targeted efforts to be sought and assessed. This study suggests that appropriate connectivity measures and standardized practice guidelines, and simplified platforms could address clinicians’ concerns and improve virtual care adoption.

## Supporting information

S1 Data Virtual visits during COVID-19 survey data. (XLSX)

S1 File Survey questionnaire. (DOCX)

S1 Fig Distribution of responses by OHT. (DOCX)

S2 Fig Platforms used to offer virtual visits. (DOCX)
