# Supplementary material for: Exploring the use and challenges of implementing virtual visits during COVID-19 in primary care and lessons for sustained use
Source: PLoS One. 2021 Jun 24;16(6):e0253665. doi: 10.1371/journal.pone.0253665 (PMC8224904; doi:10.1371/journal.pone.0253665)
Supplement: S1 File — (DOCX) [file pone.0253665.s002.docx]

**S1 File. Survey questionnaire.**

# Virtual visits

1. Do you currently offer any virtual visits? (e.g. telephone, email, secure messaging, text messaging or video appointments)

☐ Yes

☐ No

1. What kind of virtual visits do you offer? *Check all that apply.*

☐ Phone call

☐ Video visits

☐ Secure messaging (i.e. encrypted, PHIPA-compliant messaging platform)

☐ Regular e-mail (unsecured/non-encrypted)

☐ Text messaging

☐ Other (please describe): _________________________

1. Thinking about all the appointments in the past week, what proportion of your patient visits do you **currently** conduct virtually (phone, video, secure messaging, e-mail, text messaging)?

Sliding scale: 0% – 100%

1. In general, what proportion of your patient visits did you conduct virtually **before** COVID-19?

Sliding scale: 0% – 100%

1. In general, what proportion of **future visits** (post-pandemic) do you anticipate you will conduct virtually?

Sliding scale: 0% – 100%

1. If you are currently offering virtual visits, what products or platforms do you use? *Check all that apply.*

☐ Adracare

☐ Cloud DX

☐ Doxy.me

☐ Facetime (Apple)

☐ Google Hangouts

☐ Health Myself

☐ InputHealth Collaborative Care Record

☐ Insig

☐ Livecare (CloudMD)

☐ Medeo

☐ MediSeen

☐ Novari eVisit

☐ OnCall Health

☐ OTN Direct-to-Patient Video Visits (eVisits)

☐ REACTS

☐ Skype

☐ Teams (Microsoft)

☐ TELUS EMR Video Visits

☐ ThinkResearch VirtualCare

☐ VirtualClinic+

☐ WellX

☐ Zoom.us (free non-secure version)

☐ Zoom.us (paid secure healthcare version)

☐ Other: _______________________

1. What is your comfort level with using different modes of virtual visits?

|  | **Very Uncomfortable** | **Uncomfortable** | **Neutral** | **Comfortable** | **Very Comfortable** | ***Not Applicable/ Not Using*** |
| --- | --- | --- | --- | --- | --- | --- |
| Phone call | ☐ | ☐ | ☐ | ☐ | ☐ | ☐ |
| Video visits | ☐ | ☐ | ☐ | ☐ | ☐ | ☐ |
| Secure messaging | ☐ | ☐ | ☐ | ☐ | ☐ | ☐ |
| E-mail (unsecured) | ☐ | ☐ | ☐ | ☐ | ☐ | ☐ |
| Text messaging | ☐ | ☐ | ☐ | ☐ | ☐ | ☐ |

1. How well were you able to integrate virtual visits within your day to day workflow?

☐ Very well

☐ Moderately well

☐ Poorly (please explain) ________________________

1. What is your overall experience with offering virtual visits?

☐ Very satisfied

☐ Satisfied

☐ Neither satisfied nor dissatisfied

☐ Dissatisfied

1. What are the barriers/challenges that you experience with virtual visits? *Check all that apply.*

☐ Unable to justify cost

☐ Concerns about patient privacy

☐ Obtaining e-mail consent

☐ Concerns about increase in demands on time

☐ Lack of integration with current workflow or EMR

☐ Adequate training/education

☐ Adequate administrative support (e.g. comfort with technology, not enough administrative staff to delegate tasks, etc.)

☐ Connectivity issues (e.g. inconsistent Wi-Fi/Internet connection)

☐ Concerns about patients overusing services

☐ Patients’ limited access to technology/devices (e.g. computer/laptop/tablet, phone app)

☐ Patients’ limited technical knowledge

☐ Other (please specify): _____________________

1. Are there any supports you accessed and found useful to help you with integrating virtual visits in your practice? *Check all that apply.*

☐ Local colleague support (Connection with a local colleague who is using the technology)

☐ In-house organizational supports (e.g. IT support, Data/EMR Administrators, Quality Improvement Specialists, etc.)

☐ Change management supports (e.g. workflow integration, defining roles in the team)

☐ Technical training on how to use tool (webinars, recorded videos, one-on-one support etc.)

☐ Written information about how to integrate the tool into workflow

☐ Evidence about the effectiveness of the tool

☐ Virtual care standards outlined by my profession’s college

☐ Written resource on comparison of virtual visit platforms (cost, features, pros/cons etc.)

☐ Other (please specify): _____________________________

# Demographic Information

While the following questions are optional, your responses will help us better understand the results. Please answer what you are comfortable with sharing.

1. What is your practice size?

☐ Solo physician/practitioner

☐ Small group (2-3 physicians)

☐ Medium group (4-10 physicians)

☐ Large group (11+ physicians)

1. Please indicate your practice geography type.

☐ Urban

☐ Rural

1. Please indicate the Ontario Health Team (OHT) that your practice would be affiliated with.

| - Brantford Brant OHT |
| --- |
| - Burlington OHT |
| - Cambridge North Dumfries OHT |
| - Chatham Kent OHT |
| - Elgin County OHT |
| - Grey Bruce OHT |
| - Guelph and Area OHT |
| - Haldimand OHT |
| - Hamilton OHT |
| - Huron and Perth OHT |
| - KW4 OHT (Kitchener, Waterloo, Wellesley, Wilmot, and Woolwich) |
| - Niagara OHT |
| - Oxford OHT |
| - Rural Wellington OHT |
| - Sarnia Lambton OHT |
| - Western OHT |
| - Windsor Essex OHT |

1. What is your gender?

☐ Male

☐ Female

☐ Prefer not to disclose

☐ Other: _______________

1. How many years have you been in practice in Canada? _________________
2. How long have you been using EMRs in general?

☐ Under 1 year

☐ 1-5 years

☐ 5-10 years

☐ 10+ years

1. Please indicate your comfort level with your current EMR?

☐ Novice/Beginner

☐ Average

☐ Expert/Advanced

1. What is your overall comfort level with using technology in general?

☐ Novice/Beginner

☐ Average

☐ Expert/Advanced
