# Supplementary figures and images for: Exploring the use and challenges of implementing virtual visits during COVID-19 in primary care and lessons for sustained use
Source: PLoS One. 2021 Jun 24;16(6):e0253665. doi: 10.1371/journal.pone.0253665 (PMC8224904; doi:10.1371/journal.pone.0253665)

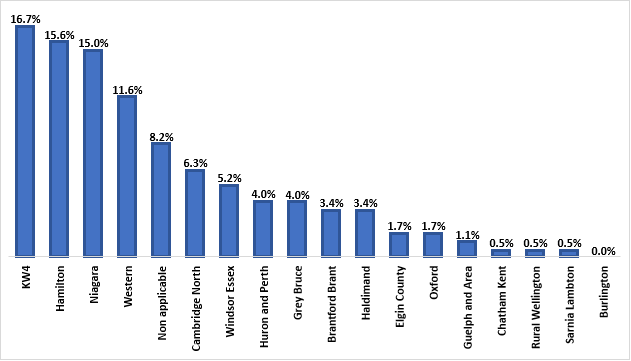


**S1 Fig. Distribution of responses by OHT.**

Supplement: S1 Fig — (DOCX) [file pone.0253665.s003.docx]
