# Supplementary material for: Exploring the use and challenges of implementing virtual visits during COVID-19 in primary care and lessons for sustained use
Source: PLoS One. 2021 Jun 24;16(6):e0253665. doi: 10.1371/journal.pone.0253665 (PMC8224904; doi:10.1371/journal.pone.0253665)
Supplement: S2 Fig — (DOCX) [file pone.0253665.s004.docx]

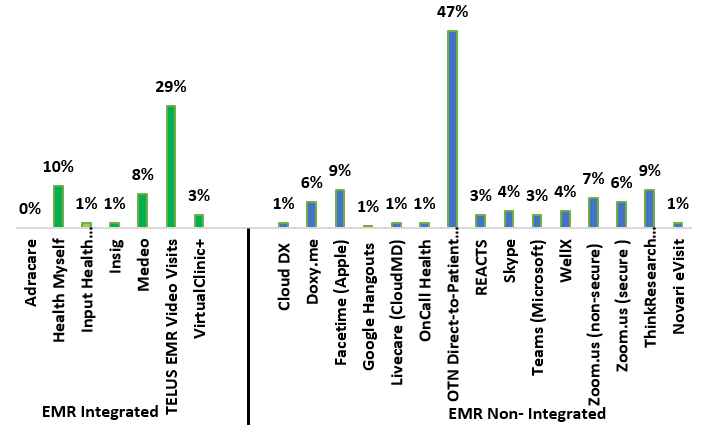


**S2 Fig. Platforms used to offer virtual visits**

*Participants were invited to choose all that apply for the questions
